# Effect of High-Dose Vitamin C Infusion in a Glucose-6-Phosphate Dehydrogenase-Deficient Patient

**DOI:** 10.1155/2017/5202606

**Published:** 2017-11-26

**Authors:** Joseph Quinn, Bryan Gerber, Ryan Fouche, Katharine Kenyon, Zachary Blom, Purushothaman Muthukanagaraj

**Affiliations:** ^1^Departments of Emergency Medicine, Internal Medicine, and Critical Care Medicine, East Carolina University, Greenville, NC, USA; ^2^Departments of Emergency Medicine and Internal Medicine, East Carolina University, Greenville, NC, USA; ^3^Department of Internal Medicine, East Carolina University, Greenville, NC, USA; ^4^East Carolina University, Greenville, NC, USA; ^5^Departments of Internal Medicine and Psychiatry, East Carolina University, Greenville, NC, USA

## Abstract

Vitamin C supplementation is generally regarded as benign. There has been a resurgence of interest in the general medical community regarding the use of vitamin C most notably in the care of sepsis. Nonetheless, caution must be taken if supraphysiologic vitamin C supplementation is being administered as it should be considered a medication just like any other. We present a case of hemolysis in a glucose-6-phosphate dehydrogenase- (G6PD-) deficient patient receiving high-dose vitamin C infusions for his rheumatoid arthritis.

## 1. Introduction

Vitamin C is a water-soluble vitamin that is generally regarded as a benign medical intervention if supplemented. Though the benefits of vitamin C have been extolled for many years, there has been a resurgence of interest in the general medical community regarding the use of vitamin C most notably in the care of sepsis. For most patients, supplementing with vitamin C is benign. However, there remain patient populations for which caution must be used if vitamin C supplementation is being administered at levels that are considered supraphysiologic. We present a case of hemolysis in a glucose-6-phosphate dehydrogenase- (G6PD-) deficient patient receiving vitamin C infusions for his rheumatoid arthritis.

## 2. Case Presentation

A 59-year-old African American male with a past medical history of chronic lymphocytic leukemia (CLL)/small lymphocytic lymphoma (SLL), hypertension, and rheumatoid arthritis presented from an outside facility with symptomatic anemia and white blood cell elevation that was noted to be predominately lymphocytic. He had a known history of CLL/SLL via lymph node biopsy that was diagnosed three months prior to admission. At the outside facility, he was noted to be hypoxic and required four liters of oxygen/minute to maintain oxygen saturation above 85%. An arterial blood gas (ABG) was performed demonstrating a pH of 7.53, pCO_2_ of 32 mmHg, and a PaO_2_ of 550 mmHg while on 4 L/min of oxygen. Methemoglobin and carboxyhemoglobin levels were automatically drawn as part of the ABG assay. This revealed a methemoglobinemia of 5.9% and a carboxyhemoglobin elevation of 4%. Other labs demonstrated a hemoglobin level of 5.9 g/dL with an elevation of his white blood cell count to 53.10 k/uL. He was also noted to have an elevated lactate dehydrogenase and reduced haptoglobin level concerning for intravascular hemolysis. Due to his history of CLL/SLL, there was a concern that his anemia was due to CLL marrow infiltration or an autoimmune hemolytic anemia. The patient was subsequently transferred to our facility for further management.

Upon interview, he endorsed three days of shortness of breath with associated weakness and dizziness prior to admission to the outside facility. He also noted that he had some associated back pain with very dark urine. In light of his elevated carboxyhemoglobin and methemoglobin levels, we asked him social and exposure questions. He denied smoking, recent exposure to exhaust or fire, local anesthetics, and topical or oral numbing medications. Interestingly, he noted that he had a very similar episode four months prior after receiving an IV dose of vitamin C for his rheumatoid arthritis that had been uncontrolled with biologics. At that time, he received a single dose of packed red blood cells and demonstrated clinical improvement. He was discharged home after stabilization of his hemoglobin. He stated that, prior to this most recent episode, he had received 75 grams of intravenous high-dose vitamin C. He noted that, within an hour of the infusion, he started to develop shortness of breath.

Upon arrival to our facility, the patient was started on high-dose prednisone as the concern was that his CLL/SLL could have caused the hemolysis. While his lymphocytosis did decrease on the steroids, dropping from 53.1 k/uL to 33.3 k/uL, he continued to have hemolysis. He developed acute kidney injury thought to be secondary to hemolysis that resolved with fluid administration. His course was rather uncomplicated with a total of three units of packed red blood cells transfused through a five-day inpatient stay to compensate for his continued hemolysis even in the setting of improved lymphocytosis. His hemoglobin eventually stabilized, and he was discharged home. A G6PD screen was performed during his stay, and this was negative for deficiency. As there was a strong suspicion of a G6PD deficiency due to the onset of hemolysis and symptoms after high-dose vitamin C infusion with resultant methemoglobinemia, we requested records from the physician who administered the high-dose vitamin C. These records revealed that two G6PD assays prior had demonstrated low G6PD levels.

## 3. Discussion

Pharmacologically dosed vitamin C has been used for chronic illnesses including hypertension, cancer, arthritis, Parkinson's disease, and innumerable other diseases for the past forty years [1]. The perceived benefits of this intervention outweigh the perceived risks as vitamin C is generally considered a benign intervention in most clinicians' minds. Nonetheless, caution needs to be exercised with high-dose vitamin C in patients with G6PD deficiency.

G6PD deficiency is a hereditary X-linked recessive disorder with an estimated worldwide prevalence of at least 329 million people. Prevalence varies among different geographical regions and ethnicities. Because of the X-linked nature of this disorder and subsequent X-chromosomal inactivation of females, the clinical phenotype is relatively variable. As a result, most prevalence studies are estimated based on male subjects as the phenotypic presentation is more stable [2]. When broken down by race, African Americans (10.2%) and Asians (3.6%) were more likely to have G6PD deficiency than Hispanics or Caucasians [3]. There are over a hundred identified mutations of the G6PD gene, resulting in differing phenotypes with variable amounts of enzyme deficiency. The majority of patients with G6PD deficiency remain asymptomatic throughout their lives. Males are disproportionately affected when serious clinical consequences occur because of the X-linked nature of the disease. When the disease becomes clinically apparent, the two most common manifestations are neonatal jaundice and hemolytic anemia after exposure to oxidizing medications or infections [2].

G6PD catalyzes the first step of the pentose phosphate pathway, one function of which is to protect cells against oxidative damage [2, 4]. By reducing nicotinamide adenine dinucleotide phosphate (NADP) to nicotinamide adenine dinucleotide phosphate hydrogen (NADPH), G6PD allows the cells to maintain glutathione in its reduced form. This in turn allows glutathione peroxidase to function properly and rid the cells of peroxide [4]. Erythrocytes rely exclusively on the pentose phosphate pathway and therefore G6PD to provide NADPH for this purpose since they lack mitochondria [2]. The rate of G6PD activity is controlled by the concentrations of NADPH and NADP in the cell. When NADPH is oxidized to NADP, G6PD activity increases to replenish NADPH [4]. In G6PD deficiency, erythrocytes are limited in their ability to increase G6PD activity and are, therefore, more vulnerable to oxidative damage [2]. Though there can be an element of extravascular hemolysis in G6PD deficiency with the formation of Heinz bodies from denatured hemoglobin passing through the reticuloendothelial system [3], the primary hemolysis in G6PD deficiency is intravascular and accounts for the increased LDH and decreased haptoglobin seen in the patient [5].

In this case, the patient's ABG was helpful in diagnosis and was the lab abnormality that prompted the team to suspect G6PD deficiency as the underlying cause of hemolysis. Carboxyhemoglobin is often caused by various exposures but can also be caused by increased endogenous production of carbon monoxide. Among the major causes of increased endogenous production is that of increased heme catabolism. In most cases of hemolysis, increased levels of carboxyhemoglobin are to be expected and can correlate with the degree of hemolysis [6]. In addition to the increase in carboxyhemoglobin, our patient also had elevated methemoglobin. Elevated methemoglobin levels, similar to carboxyhemoglobin, can be caused by various exposures and are usually caused by exposure rather than hereditary methemoglobinemia. Methemoglobin rises when heme is oxidized to the ferric form (Fe^3+^) from the normal ferrous (Fe^2+^) [7]. In G6PD deficiency, the inability to protect against oxidative damage can lead to methemoglobinemia. The enzyme that reduces methemoglobin, NADH-cytochrome b_5_ reductase, normally keeps this value at less than 1%, but in cases of acute oxidative stress, this can be overwhelmed as was seen with the patient's increased methemoglobin level [8].

The gold standard for diagnosis of G6PD deficiency is mainly the one performed by biochemical quantitative analysis using spectrophotometry [9]. Rapid screening spot tests have allowed easier and faster access to determine G6PD status [2]. More specifically, when viewed under ultraviolet lighting, the lack of fluorescence of NADPH being generated from NADP in the pentose-phosphate pathway provides a positive finding qualitatively [2, 10, 11]. If suspected, however, one must avoid the rapid fluorescent spot test during times of acute hemolysis as the qualitative screen can give a false-negative interpretation [12]. The process of hemolytic anemia causes degradation of aged erythrocytes with lower levels of G6PD. The resultant reactive reticulocytosis that occurs during this hemolysis creates new erythrocytes that have normal levels of G6PD initially [13]. In our case, the G6PD screen during his admission while acutely hemolyzing was normal. Our clinical suspicion was great enough based on history, and his underlying methemoglobinemia that inquiry into the patient's medical records from his various outside providers was made confirming two G6PD enzyme assays below normal limits.

Various etiologies can provoke an acute hemolytic episode in patients with G6PD deficiency. The end result is oxidative stress within the red blood cells. Most documented causes of hemolysis are either from food or medication ingestion. Common medications that are cited include dapsone, methylene blue, nitrofurantoin, primaquine, rasburicase, and NSAIDs [14]. Classic food items include fava beans and other legumes, red wine (sulfites), tonic water (quinine), vitamin K, and menthol. It is difficult to identify many drug interactions that may lead to hemolysis from in vitro studies, as metabolic byproducts can lead to hemolysis. Additionally, the high variability of G6PD deficiency, particularly in females due to X-chromosomal inactivation, can present with variable amounts of hemolysis [15]. Other documented causes of hemolysis in G6PD-deficient individuals include infections [16], DKA [17], and hypoglycemia [18].

Ascorbic acid is well known as an antioxidant as it is able to donate an electron to reduce oxidizing radicals and may be protective in G6PD deficiency at slightly supraphysiologic levels [19]. Though high doses of vitamin C have been shown to cause little sequela in healthy patients [20], case reports have documented high doses of vitamin C causing hemolysis in G6PD-deficient patients [1, 21, 22]. Studies have shown that high doses of ascorbic acid decrease the function and survival of G6PD-deficient red blood cells as well [23]. High-dose ascorbic acid is thought to promote the production of hydrogen peroxide as a by-product of its cycling between its ionized form and the ascorbate radical form [24]. Hydrogen peroxide is a potent oxidizer, and increased production results in damage of G6PD-deficient RBCs and ultimately hemolysis in these patients. The enzyme glutathione peroxidase needs reduced glutathione to help eliminate hydrogen peroxide. As mentioned, the decrease in NADPH decreases the overall storage of reduced glutathione and essentially renders the glutathione peroxidase ineffective [4]. Our patient's immediate symptoms after both doses of vitamin C infusion implicated vitamin C-associated hemolysis in light of his low level of G6PD that had been measured in the past.

In a recent retrospective before and after study by Marik et al., early treatment with high-dose intravenous vitamin C combined with corticosteroids and thiamine showed promising results, leading to reduced end-organ damage and mortality in severe sepsis and septic shock with reduced ICU length of stay [25]. While further investigation is required for validation, such a treatment protocol has the potential to be widely adopted based on how benign the underlying interventions are perceived. Care must be exercised in certain groups, as the resultant hemolysis from high-dose vitamin C could be misconstrued as worsening sepsis rather than an iatrogenic insult in a G6PD patient. This could lead to continuing a therapy that could be potentially fatal in a select group of patients.

## 4. Conclusion

Our case showed that G6PD deficiency should be considered any time hemolytic anemia ensues after a medication is administered. Additionally, our case highlighted that an acute hemolytic anemia associated with methemoglobinemia by no means diagnostic could lead the clinician to a potential diagnosis of G6PD deficiency with large amounts of hemolysis. Finally, our case exemplified the importance of understanding the limitation of qualitative testing in an acute hemolytic reaction. Though supraphysiologic vitamin C is considered benign in most people, it can be associated with major morbidity and even mortality in G6PD-deficient patients. Care must be taken to remember that, when administered at high doses, vitamin C is a medication with side effects just like any other medication one would prescribe. For the clinician who will be utilizing vitamin C at supraphysiologic doses, screening should be considered prior to utilization, particularly in ethnicities with a higher prevalence, to avoid potential false negatives during treatment and, more importantly, iatrogenic harm to the patient.
